# Can Isoflavone-Rich Legume Plants Be Useful in the Chemoprevention of Hormone-Dependent Cancers?—A Systematic Review

**DOI:** 10.3390/ijms25137389

**Published:** 2024-07-05

**Authors:** Wojciech Paździora, Paweł Paśko, Karolina Grabowska, Agnieszka Galanty

**Affiliations:** 1 Department of Pharmacognosy, Jagiellonian University Medical College, Medyczna 9, 30-688 Kraków, Poland; wojciech.pazdziora@doctoral.uj.edu.pl (W.P.); karolina1.grabowska@uj.edu.pl (K.G.); 2 Doctoral School of Medical and Health Sciences, Jagiellonian University Medical College, 16 Łazarza Str., 31-530 Cracow, Poland; 3 Department of Food Chemistry and Nutrition, Jagiellonian University Medical College, Medyczna 9, 30-688 Kraków, Poland; p.pasko@uj.edu.pl

**Keywords:** chemoprevention, breast cancer, prostate cancer, isoflavones, *Fabaceae*

## Abstract

Plants from the *Fabaceae* family are widely distributed around the world, especially in Europe, Asia and North America. They are a rich source of isoflavones, compounds with estrogen-like activity, which are suspected of having a chemopreventive effect against hormone-dependent cancers. Following the PRISMA guidelines, we conducted a systematic review aimed at assessing the impact of *Fabaceae* plant extracts on hormone-dependent cancer cells and the content of active compounds in plant raw materials. We analyzed the results of 63 articles from in vitro and in vivo studies describing the effect of plant extracts containing isoflavones on cancer cells, along with their anti-inflammatory and antioxidant potential. In the process, we determined the research limitations and future research directions. The collected results indicate the plant species with potentially high contents of phytoestrogens and anti-inflammatory, antioxidant and cytotoxic properties. They point to the potential use of plants in the diet as a source of compounds offering cancer prevention.

## 1. Introduction

Chemoprevention is the use of substances of natural or synthetic origin in order to inhibit, reverse or delay the process of carcinogenesis. The idea was created by American pharmacologist Dr. Michael Sporn in the 1970s, who believed that the search for new drugs should be focused both on cancer prevention (chemoprevention) and treatment (chemotherapy). This process is multidirectional and involves many levels of cellular metabolism [1]. The first element is the inhibition of carcinogen activation by stopping the expression or activation of cytochrome P450. The detoxification process is one of the critical stages in which the body, trying to neutralize the procarcinogen using appropriate enzymes and proteins, activates cytochrome P450 into a carcinogen that may have a negative effect on healthy cells. It is also important to stimulate carcinogen detoxification by inducing enzymatic activity, e.g., glutathione S-transferases, epoxide hydrolase or heme oxygenase-1 [2]. Enzymatic induction is possible due to the ability of chemopreventive compounds to break the bond between the nuclear erythroid 2-related factor (Nrf2) and the cytoskeleton protein Kelch-like ECH associating protein (Keap1). Nrf2 accumulates in the cell nucleus and activates many cytoprotective genes [3,4]. Other important features, expected from chemopreventive substances, are the ability to stop the cell cycle in any phase and induce apoptosis by stimulating, among others, caspases (mainly caspase-3) and BAX protein and inhibiting the expression of the anti-apoptotic protein BCL-2. Moreover, cell-protective processes also include the anti-inflammatory effect, through the suppression of pro-inflammatory factors (e.g., cytokines) or enzymes facilitating the inflammation process (e.g., hyaluronidase, elastase). Finally, antioxidant properties are also crucial elements of the chemopreventive strategy, allowing for protection against an excess of free radicals and related cell damage [5]. Examples of natural compounds with chemopreventive effects include carotenoids, flavonoids, proanthocyanidins, isothiocyanates and omega-3 fatty acids [6].

**Figure 1:**
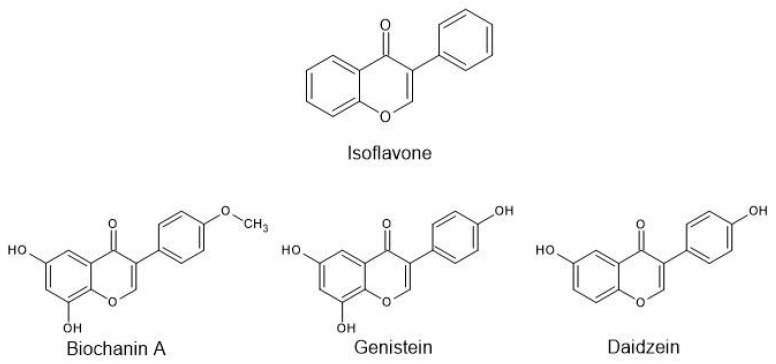
Examples of structures of the most commonly known isoflavones.

Isoflavones (Figure 1) are a special subclass of flavonoids with a structural similarity to female sex hormones—estrogens—and thus the ability to bind to estrogen receptors (ERs) in the cell nucleus. The presence of hydroxyl groups in the chemical structure, especially in the 5′ and 7′ positions of the ring, determines the biological activity towards this receptor, due to the structural similarity to 17-β-estradiol. Isoflavones, often termed phytoestrogens, also compete for the binding site with endogenous estrogen, increasing its level in the serum. One of the compounds belonging to this group is genistein, which at a concentration of 100 nM can induce a biological effect similar to 17-β-estradiol at its physiological concentration [7].

**Figure 2:**
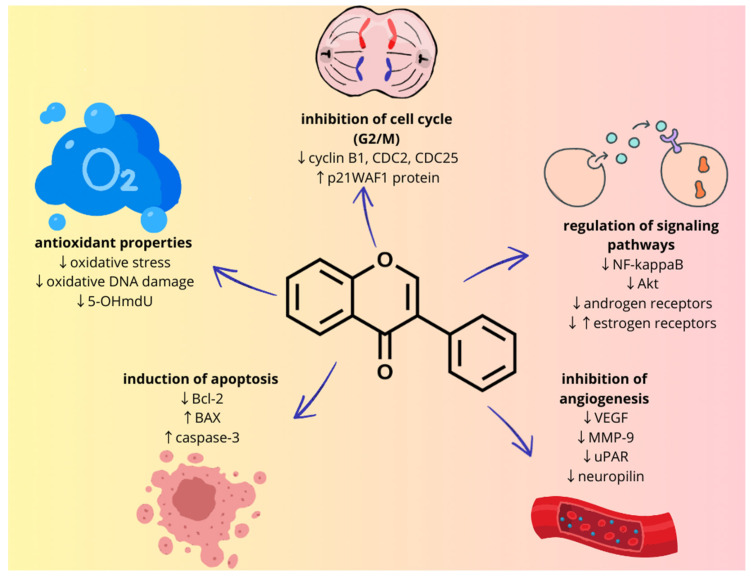
Molecular mechanisms of isoflavone chemoprevention. ↑ increase, ↓ decrease.

The chemopreventive effects of isoflavones include a direct impact on cancer cells’ functioning, anti-inflammatory or antioxidant effects and inhibition of angiogenesis (Figure 2). Genistein and daidzein inhibit tyrosine-specific protein kinases, which are among the elements that determine cancer cell proliferation. Genistein, daidzein and biochanin A are cell cycle inhibitors and induce apoptosis in some cancer cells [8]. Moreover, as with other polyphenolic compounds, isoflavones are characterized by antioxidant properties. Neutralization of free hydroxide and peroxide radicals is one of the main mechanisms of the antioxidant activity of these compounds. In addition, they have metal-chelating properties, especially divalent cations, and stimulate antioxidant enzymes such as superoxide dismutase, glutathione reductase and many types of catalases. Isoflavones also have an anti-inflammatory effect, as another important element of chemoprevention. In the in vitro studies on human chondrocyte cells, genistein reduced the levels of COX-2 and NO, but without affecting COX-1. Studies on mice showed that these compounds reduced the number of leukocytes in the blood and the production of interleukin 1 and 6, nitric oxide and prostaglandin E2. In human studies, consumption of soybeans, rich in isoflavones, resulted in a decrease in IL-18 and C-reactive protein [9]. Another mechanism of action of genistein and daidzein is the inhibition of the formation of TNF-α and the monocyte chemoattractant protein-1 induced by it in human umbilical vein endothelial cells [10]. Moreover, soy isoflavones influence epigenetic mechanisms, including DNA methylation, histone modification and non-coding RNA expression, and thus modulate the expression of pro-cancer genes. However, it should be mentioned that most studies have focused on specific compounds belonging to isoflavones (Pudenz 2014). An example is genistein, which decreased the levels of DNA methyltransferases (DNMT1, DNMT3a and DNMT3b) in breast cancer cell lines (MCF7 and MDA MB 231) [11]. Moreover, genistein and daidzein reduced the methylation of genes involved in DNA repair (BRCA1, GSTP1 and EPHB2) in prostate cancer cell lines (DU-145 and PC-3) [12].

The Fabaceae family includes approximately 18,000 species distributed all over the globe. Specific secondary metabolites for the Fabaceae family are isoflavones, and it has been suggested that these compounds are chemotaxonomic markers. Carvalho et al. in a recent review noted the presence of 240 different isoflavones in 69 species of this family [13]. One of the most commonly known representatives of the family is soy, particularly rich in isoflavones, the consumption of which can be considered a means of the prevention of cardiovascular diseases, hormone-dependent cancers, osteoporosis and perimenopausal syndrome [14]. In turn, the presence of isoflavones in clover, another important species with health-beneficial properties, is responsible for its estrogenic, antioxidant and cholesterol-lowering properties [15]. Plants from the Fabaceae family are generally considered safe, with the only suggestion of side effects related to the presence of anti-nutritive compounds (e.g., lectins, saponins, phytic acid) in some beans [16,17]. It should be noted, however, that there are reports of pro-cancer properties of isoflavones, as well as a possible increase in metastasis of existing cancer. Martinez-Montemayor et al. in a study on mice demonstrated a dual effect of individual soy isoflavones. Daidzein increased the growth of existing mammary tumors while increasing metastasis to the lungs and heart, while genistein reduced both tumor size and metastasis to the bones and liver. Concomitant administration of both compounds had no effect on tumor growth but increased metastasis to all the organs studied [18]. Further studies should therefore be conducted to explain these differences in action, taking into account the small differences in molecular structure.

The aim of this systematic review was to take a closer look at the less-known representatives of the Fabaceae family in terms of their chemopreventive potential and to preselect the most promising species based on the so-far-published in vitro and in vivo studies in relation to the isoflavone content.

## 2. Materials and Methods

**Figure 3:**
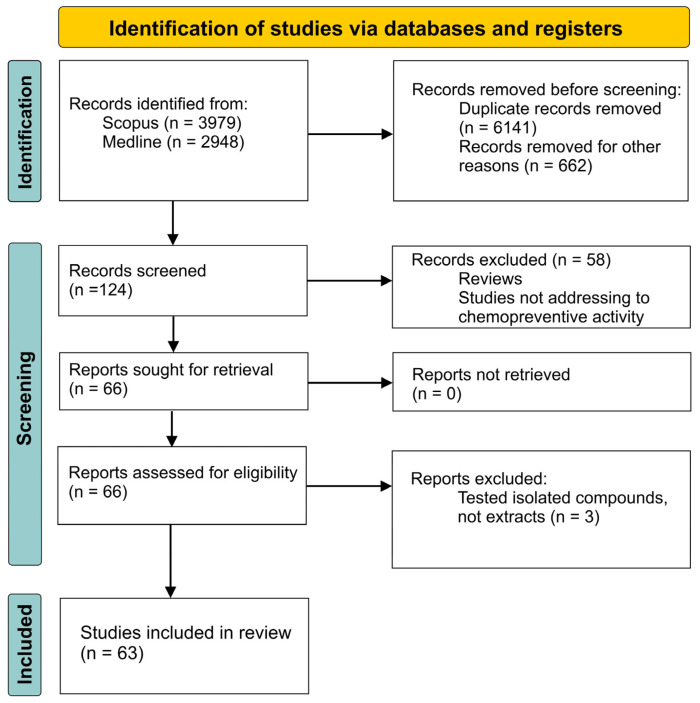
Flowchart of the search strategy.

The Medline and Scopus databases were searched, with data collected until February 2024. The keywords used were “name of genus AND isoflavones”, “name of genus AND cancer”, “name of genus AND isoflavones AND cancer”. There were no time or language limits. The inclusion criteria were original articles describing the effect of extracts from plants from the Fabaceae family on breast, ovarian, prostate, cervical and thyroid cancer cell lines, as well as anti-inflammatory and antioxidant effects. We considered in vitro and in vivo animal studies. The exclusion criteria were review articles, studies on humans, in silico studies and studies on sprouts and isolated compounds. We also excluded the data on soy, as a number of reviews and meta-analyses exist on the activity of the species. The details of the search method are presented in Figure 3. In the first stage of the search, 6927 articles were selected. Then, we began a manual selection process. Following this, 286 articles were used for further screening, and, finally, 63 articles were selected for this systematic review. This systematic review was conducted in adherence to the PRISMA (Preferred Reporting Items for Systematic Reviews and Meta-analyses) statement and registered in the Open Science Framework (OSF), with the registration code osf.io/d8pfv (https://doi.org/10.17605/OSF.IO/A8395, access date 28 June 2024).

## 3. Results

### 3.1. Astragalus sp.

The genus *Astragalus* includes almost 2500 species distributed all over the world, especially in the temperate climates of the northern hemisphere [19]. Research by Butkute et al. suggested a variable content of isoflavones in the aboveground parts of *A. glycyphyllos*, depending on the stage of plant development. In the vegetative state, the amounts of formononetin, biochanin and genistein were 9.24 ± 0.7, 8.81 ± 0.8 and 2.23 ± 0.3 mg/100 g dry matter, respectively, while in the flowering state, these values were 5.50 ± 0.6, 3.17 ± 0.4 and 5.71 ± 0.6 mg/100 g dry matter. Moreover, the highest content of formononetin was noted in the stems and the lowest in the flowers [20]. In different varieties of *A. membranaceus*, in addition to the commonly occurring isoflavones, glycosides such as ononin and calycosin were found, and the values of individual isoflavones ranged from 0.0035 to 0.733 mg/g d.m. [21].

#### 3.1.1. Cytotoxic Activity

The only study to date assessing the impact of the plants from *Astragalus* sp. on hormone-dependent cancer cells concerns *A. oocephalus* L. The leaf extracts had moderate cytotoxic activity on MCF-7 breast cancer cells, with IC_50_ 74.72 ± 5.20 μg/mL [22]. No data exist on the in vivo chemopreventive effect of *Astragalus* plants.

#### 3.1.2. Antioxidant Potential

*Astragalus cicer* L. and *A. glycyphyllos* L. are two species to have been assessed for their antioxidant capacity. Despite the absence of reference substances, it can be noted that *A. cicer* showed higher activity in the DPPH test (IC_50_ 128.6 μmol/g d.m.), while the value for *A. glycyphyllos* was 35.64 μmol/g d.m. In the iron ion chelation capacity test, the results were similar for both species at about 10 μmol/g d.m. [20].

### 3.2. Cytisus sp.

The genus *Cytisus* includes 30 species, naturally occurring in Europe (except the Caucasus, Finland and northern Russia) and north-eastern Africa. The most well-known compounds in the plant are quinoliziide alkaloids, such as sparteine and sarotaminine. However, the presence of isoflavones is also notable. According to Hangau et al., the aerial parts of *C. albus* may offer a source of several isoflavones. This content depends on the acid hydrolysis of the extracts, which notably affects the total content of isoflavones (201.33 μg/mL before hydrolysis and 9.24 μg/mL after hydrolysis). The dominant isoflavone in the species is ononin (192.6 ng/mL), but its content was revealed only in unhydrolyzed extracts [23]. The author also analyzed the herb *C. nigricans*, but the only isoflavone detected in the extract was ononin (270.6 ng/mL) [24]. The species *C. scoparius* is characterized by a relatively high content of genistein, which in the aboveground parts reached 155 ± 1.5 mg/kg d.m. The values of the remaining isoflavones present in the plant did not exceed 40 mg/kg d.m. [25].

#### 3.2.1. Cytotoxic Activity

Several in vitro studies have been performed using extracts from two *Cytisus* species—*C. villosus* and *C. multiflorus*. Bouziane et al. compared the effects of aqueous and ethyl acetate extracts from *C. villosus* aerial parts on MCF-7 and T47D human breast cancer cell lines, in the concentration range of 0.5–10 mg/mL. MCF-7 and T47D cells were more sensitive to ethyl acetate extract, with LD_50_ values of 2.2 ± 0.1 and 1.57 ± 0.06 mg/mL, respectively, compared to aqueous extract (2.6 ± 0.1 and 3.8 ± 0.2 mg/mL, respectively). A dose-dependent cytotoxic effect was also observed, with the concentration of 5 mg/mL being the most effective. However, it is worth noting that the analysis of the chemical composition of the extract did not reveal the presence of isoflavones [26]. Garcia-Oliveira et al. conducted an assessment of the properties of *C. multiflorus* hydroethanolic extracts and an infusion from flowers. The cytotoxic effect of extracts in the concentration range of 6.25–400 μg/mL was assessed against MCF-7 human breast cancer and HeLa cervical cancer cell lines. Both cell lines were more sensitive to the infusion (GI_50_ 235.8 ± 8.5 and 133.3 ± 9.6 µg/mL, respectively), but in both cases, the effect was much lower than for ellipticine (0.91 ± 0.04 and 1.91 ± 0.06 µg/mL, respectively), used as a reference [27]. None of these studies associated the cytotoxic effect with the content of isoflavones in the plant. However, the authors tried to attribute the cytotoxic effect to specific groups of compounds contained in the extracts. Bouziane et al. explained the effect by the content of polyphenolic compounds, including myricetin, quercetin, epigallocatechin and kaempferol, and Garcia-Oliveira et al. by the presence of phenylethanoid glycosides.

#### 3.2.2. Anti-Inflammatory Activity

In vitro and in vivo investigations of the anti-inflammatory properties of *Cytisus* species were also carried out. In the study mentioned earlier conducted by Garcia-Oliveira et al., anti-inflammatory effects were assessed by using RAW 264.7 macrophages treated with lipopolysaccharide (LPS) to induce inflammation. The obtained IC_50_ values of the *C. multiflorus* ethanol extract (>400 (µg/mL)) and infusion (293.2 ± 11.8 µg/mL) were significantly different from the effect of the reference drug dexamethasone (6.0 ± 1.0 µg/mL), and the infusion was more effective than the alcoholic extract [27].

The only in vivo study used an aqueous extract of *C. villosus* (500 mg/kg) from leaves administered orally in Wistar rats, examining its anti-inflammatory and anti-edema effects. The carrageenan-induced paw edema decrease after 6 h in the group treated with the plant extract was comparable with the effect of indomethacin at the dose of 10 mg/kg body weight (b.w.) (77.49% ± 0.59). In the qualitative analysis of the extract, daidzin was the only isoflavone detected [20]. The authors of the study did not link the observed activity with a specific component of the extract.

#### 3.2.3. Antioxidant Potential

Antioxidant properties of various extracts of *Cytisus* sp. were previously tested, including ethanol, water, ethyl acetate and an infusion (Table 1). The most commonly used methods for assessing the antioxidant activity were the ABTS and DPPH tests. These tests are based on the ability of the tested extracts to inhibit free radicals, respectively, DPPH (DPPH•) (2,2-diphenyl-1-picrylhydrazyl) and ABTS (ABTS•+) (2,2-azino-bis-3-ethylbenzothiazoline-6-sulfonic acid). In most studies, the extracts did not show a greater oxidative potential compared to standard substances such as BHT (butylated hydroxytoluene) or ascorbic acid. The exceptions were the water and alcohol extracts from *C. multiflorus*, which were more effective than the reference Trolox sample, in the thiobarbituric acid reactive substance assay.

### 3.3. Dorycnium sp.

The genus includes 12 species found mainly in the Mediterranean Sea and Macaronesia [29]. The information on the isoflavones in the *Dorycnium* species is limited to one study, indicating low amounts of genistein, with its maximum content of 0.52 μg/g determined before acid hydrolysis [23].

#### Cytotoxic Activity

An extract from *Dorycnium pentapyllum* subsp. *Haussknechtii* had a cytotoxic effect on MCF-7 breast cancer cells, with an IC_50_ value of 20.08 μg/mL after 72 h of incubation. No effect on the non-cancerous MCF-12A breast epithelial cells was noted, which suggests a selectivity of the action. Moreover, the effect of the extract was time- and dose-dependent. Interestingly, after 48 and 72 h in the MCF-7 cell line, a significant increase in the concentration of malondialdehyde was observed, which suggests an intensification of lipid peroxidation processes. At the same time, a decrease in the amount of glutathione was observed 48 h after adding the extract, though within the next 24 h, the glutathione level returned to normal [30]. It is worth noting that the author did not determine the composition of isoflavones.

### 3.4. Genista sp.

The genus *Genista* includes 155 species that naturally inhabit Europe, western Asia and northern Africa. Of all the species studied, the majority of the studies on the content of isoflavones concern *Genista tinctoria*, characterized by a high content of genistein compared to other compounds like ononin, formononetin, daidzin, daidzein and genistin. The highest reported genistein content in the species was 535.21 ± 13.90 mg/g extract [31]. Interestingly, hydrolysis of the extract revealed the presence of formononetin, which was not present in the extract before hydrolysis (675.70 μg/mL) [23]. Kiss et al. optimized the extraction of *Genista* plant material using various methods, including percolation, maceration and adjustment of the volume of solvent used, mass and morphotic part of the extracted plant material. The results indicated interesting differences between the methods; for example, when using percolation (1000 mL of solvent, 57.32 g of herb), the sum of isoflavone was 827.42 µg/mL, and when using maceration (1000 mL of solvent, 142.25 g of herb), it was 999.52 µg/mL [32].

#### 3.4.1. Cytotoxic and Anti-Inflammatory Activity

In vitro studies using extracts from *Genista* sp. have focused on assessing the cytotoxic and anti-inflammatory potential (Table 2). The analyzed studies are characterized by considerable methodological diversity. Compared to other Fabaceae species, research was carried out on a significant number of *Genista* representatives (e.g., *G. acanthoclada*, *G. hassertiana*, *G. depressa*, *G. millii*, etc.), different morphotic parts (flowers, seeds, aerial parts) and a variety of human cancer cell lines, including breast, ovary, cervix and, what is worth emphasizing, also prostate. Interesting results were obtained for methanol extracts from *G. hassertiana* and *G. millii* at a dose of 1 mg/mL, where the agonist effect was equal to or greater than that of estrogen in MCF-7 and Ishikawa cells, respectively [33]. In turn, infusions with *G. tridentata* showed a greater growth-inhibitory effect on MCF-7 and HeLa cell lines than hydroethanolic extracts [28]. Very important information was provided by Bontempo et al., who demonstrated that fractions obtained from the methanol extract from *G. sessilifolia*, containing genistin and isoprunetin, arrested the cell cycle in the pre-G1 phase and were strong inducers of apoptosis, in turn, inducing the expression of TRAIL, DR5 and p21 [34]. Diaz et al. showed that PC-3 cells are more sensitive to an extract from *Genista monspessulana* than SiHa cells, and what is more, noted a dependence of the strength of the extract on the place of collection of the plant raw material [35].

In a study of the anti-inflammatory potential, conducted on RAW 264.7 macrophages stimulated with LPS, an infusion also turned out to be more effective than a hydroethanolic extract [27]. Elsewhere, the influence of extracts from various morphotic parts of *G. tridentata* on the reduction in pro-inflammatory factors was attributed to the presence of isoflavones such as genistein and daidzein [36].

#### 3.4.2. In Vivo Studies

So far, the only in vivo study assessed the antioxidant and protective potential of *G. tinctoria* against the toxic effects of bisphenol A, orally administered for 90 days to rats (n = 72). The administered extract, in which the isoflavone content was determined at the level of 35 mg/kg b.w., resulted in a reduction in the level of malondialdehyde compared to the untreated group. Importantly, a similar effect was obtained in the group taking methylparaben (250 mg/kg b.w.) instead of the extract. The extract and methylparaben showed antioxidant activity in that the amount of malondialdehyde, which is an indicator of the intensity of lipid peroxidation and the production of oxygen radicals, decreased [37].

#### 3.4.3. Antioxidant Potential

A number of studies have determined the antioxidant potential of different *Genista* species (Table 3) [27,38,39,40,41]. Interesting observations were provided by Orhan et al. [38], where an increase in the antioxidant potential was notable in a DPPH test after acid hydrolysis of extracts from *G. sandrasica* and *G. vuralii*. The values obtained for the hydrolyzed extracts were similar to those for the reference, gallic acid. In order to investigate the effect of *G. tenera* extract on the activity of cyclooxygenase-1, and thus the anti-inflammatory potential, the oxidation of TMPD with arachidonic acid was observed. The enzyme was inhibited by 47.5% for n-butanol extract at a concentration of 0.5 mg/mL. The IC_50_ value for the extract was 291.60 µg/mL, compared to 60 µg/mL for the reference, indomethacin [42].

### 3.5. Lupinus sp.

This genus contains as many as 267 species, but only 14 grow in Europe. The plants originate from North and South America and also naturally inhabit the Mediterranean basin [43]. The most common isoflavone in all analyzed species was genistein, in the range of 8.7–101 mg/kg d.m. [44], along with its derivatives. A notable change occurred during seed germination of *Lupinus albus* L., where the biosynthesis of genistein increased intensively (9.39 ± 0.81 μg/g in ungerminated seeds, 20.74 ± 2.89 μg/g in germinated seeds) [45]. This was also confirmed by Ranilla et al. [46], where, in all examined varieties of *L. mutabilus*, the content of genistein was higher in cotyledons than in hypocotyls (5.7 ± 0.1 and 1.3 ± 0.1 mg/100 g f.w., respectively). This seems to be one of the ways to increase the content of isoflavones in tested samples.

#### 3.5.1. Cytotoxic Activity

In vitro studies were carried out using ethanol extracts from only two *Lupinus* species, *L. albus* and *L. angustifolius*, but the study included shoots, roots and germinated and ungerminated seeds of the plants. The effect of the *Lupinus* extracts was examined on human breast cancer lines differing in ER expression (MCF-7, MDA-MB-231, BT20), ovarian A2780 and cervical SiHa cell lines. Ethanol extracts from *L. angustifolius* shoots and roots were tested at concentrations of 0.1–200 μg/mL. In the case of MCF-7 cells, the shoot extract was the most effective (IC_50_ 18.06 ± 4.49 μg/mL), while for BT20 cells, the opposite effect was observed—the root extract turned out to be slightly more effective than the shoot (66.86 ± 7.02 μg/mL vs. 70.27 ± 0.76 μg/mL) [47]. Andor et al. assessed the effect of seed germination on the antiproliferative potential of extracts from germinating or non-germinating seeds of *L. albus* and *L. angustifolius* against breast cancer MCF7 and MDA-MB-231, ovarian cancer A2780 and cervical cancer SiHa cell lines. The concentrations of the tested extracts were 15, 50 and 150 μg/mL. The results depended on the type of cell line. Only *L. angustifolius* extracts at a dose of 150 µg/mL inhibited the proliferation of breast cancer cell lines, and this effect was independent of germination. The SiHa cell line turned out to be more susceptible to the action of plant extracts from both species. The highest percentage of proliferation inhibition (32.53 ± 1.49%) was obtained for *L. angustifolius* at a dose of 150 μg/mL. In the case of this cell line, extracts from germinating seeds of both species revealed antiproliferative effects at a dose of 50 µg/mL. The A2780 cells were resistant within the tested concentration range. Research suggests a higher content of cytotoxic substances in germinating seeds compared to cell lines [45]. The authors of neither study found a notable cytotoxic effect with the content of isoflavonoids in the tested extracts, and only Andor et al. determined the genistein content in the extracts.

#### 3.5.2. Antioxidant Potential

Several lupine species have been analyzed for their antioxidant potential (Table 4). All available studies used the DPPH test, but due to their different units, it is difficult to compare the results of these studies.

### 3.6. Medicago sp.

*Medicago* is a widespread genus, occurring naturally throughout Europe, Asia and northern and southern Africa and also introduced to other continents. As many as 14 species have been tested for an isoflavone content: *M. sativa*, *M. arabica*, *M. arabica*, *M. doliata*, *M. minima*, *M. murex*, *M. orbicularis*, *M. polymorpha*, *M. rigidula*, *M. tornata*, *M. truncatula*, *M. lupulina*, *M. scutelata* and *M. segitalis*. Most studies have been carried out on *Medicago sativa*, where interesting and significant changes in the isoflavonoid content during drying of the plant material have been reported, though it seems the impact of the process depends on the plant cultivar. For example, the content of genistein in cultivar SARDI 7 decreased from 137.0 ± 21.1 fresh weight to almost 6.5 ± 1.2 mg/kg dry weight, while the amount of genistein increased only slightly, from 17.8 ± 4.2 fresh weight to 18.3 ± 10.7 mg/kg dry matter in cultivar Magna 804 [50]. Moreover, there are qualitative differences in the species. Butkute et al. found only formononetin and biochanin A in the Malvina variety [51]. Barreira et al. conducted a large screening study of the genus *Medicago*. The highest content of formononetin was determined in *M. arabica* (2010 ± 490 mg/kg d.w.), and that for irilone was in *M. polymorpha* (1432 ± 272 mg/kg d.w.), while in the latter species, the formononetin content was only 11 ± 1.6 mg/kg d.w. [52]. Another study comparing the contents of isoflavones in several *Medicago* species showed that the type of solvent used affects their amount in the extract. For example, the hydroalcoholic extract of *M. rigidula* contained 12.21 ± 0.10 mg/kg d.w. of daidzein, while in the alcoholic extract, the amount was 8.10 ± 0.11 mg/kg d.w., and the compound was not determined in the water extract [53].

#### 3.6.1. Cytotoxic Activity

In two subsequent studies, Asadi-Samani et al. examined the cytotoxic potential of an *M. sativa* extract on breast and prostate cancer cells. The ethanol extract used in the concentration range of 1–10 μg/mL showed differences in action. The extract was ineffective against MCF7 and MDA-MB231 breast cancer cell lines and PC-3 prostate cancer cells, where the IC_50_ was <300 µg/mL. However, it was several times more effective against low-metastatic, androgen-independent DU145 prostate cancer cells, with an IC_50_ value of 77 µg/mL [54,55].

#### 3.6.2. Antioxidant Potential

The same authors assessed the antioxidant potential of 20 plant species from different families. *M. sativa* showed the weakest effect among the tested species in the DPPH test, with a result of >300 μg/mL. The value obtained for BHT as a substance used as a standard was 120.48 ± 1.42 μg/mL [54].

### 3.7. Melilotus sp.

The genus *Melilotus* includes 19 species, widespread in Europe, Asia and northern and eastern Africa [56]. In terms of their quantitative and qualitative compositions, there are notable differences between the content of compounds and the plant raw material used for testing. The melilot herb is characterized by the presence of small amounts of genistin in the range of 0.567 ± 0.03–1.79 ± 0.1 μg/mL [24,57], which is absent from seeds. In turn, the seeds contain formononetin in an amount of 19.2 ± 2.5 μg/g dry matter. The second component determined in the seeds was coumesterol from the cumestane group, with a content several times higher than the amount of isoflavone (105.0 ± 11.1 μg/g dry matter) [58].

#### 3.7.1. Cytotoxic Activity

The cytotoxic activity of *Melilotus indicus* against the MCF-7 breast cancer cell line was tested at concentrations of 0.1–100 μg/mL, and it resulted in an IC_50_ of 80.30 ± 6.31 μg/mL. This is the only study to date assessing the cytotoxic potential of any representative of *Melilotus* sp. [22].

#### 3.7.2. Antioxidant and Anti-Inflammatory Potential

The results of the analysis of the antioxidant potential of *Mellilotus officinalis* extract may seem surprising. The study was conducted on a crude extract and nanofiltrate, obtained in the process of nanofiltration of the crude extract through a membrane under a pressure of 8 bar. The EC_50_ obtained in the DPPH test by the nanofiltrate (0.459 ± 0.04 mg/mL) showed almost twice the effect of the crude extract (0.858 ± 0.12 mg/mL), but the values obtained were weaker than those of ascorbic acid (0.0036 ± 0.0002 mg/mL). The nanofiltrate inhibited hyaluronidase more strongly (the value for the IC_50_ was 11.8 ± 0.1 μg/mL) than ibuprofen (13.7 ± 0.1 μg/mL) used as a reference. In turn, the IC_50_ values in the LOX inhibition assay were 109.4 ± 1.4 (μg/mL) for crude extract, 94.8 ± 0.4 for nanofiltrate and 69.7 ± 0.3 for ibuprofen [57].

### 3.8. Ononis sp.

The genus *Ononis* occurs in Europe, northern Africa and western Asia and includes about 86 species [59]. The roots of the species are used in traditional medicine as a diuretic, due to the high content of flavonoids [60]. The roots contain isoflavones in a range from 1.76 to 3.63 g/100 g extract depending on the species [61]. Significant differences in the quantitative and qualitative isoflavone contents were found for extracts subjected to acid hydrolysis. In the study by Benedec et al., there was a notable decrease in the ononin content and an increase in the formononetin content after hydrolysis of the *O. spinosa* extract. The highest content was recorded for ononin before the hydrolysis process, at 175.72 mg/100 g d.m. It is worth noting that the glycosides daidzin and genistin could not be determined in the hydrolyzed extracts, but daidzein appeared [62]. In raw extracts from *O. spinosa*, the highest content was determined for glycosidized pterocarpanes—maackiain and medicarpin. There is also a notable tendency for most of the isoflavones to be present in the form of glycosides. Similar results were obtained when analyzing extracts from *O. arvense* [63].

#### 3.8.1. Cytotoxic Activity

Of the many species of the *Ononis* genus, only three (*O. spinosa*, *O. hirta*, *O. natrix*) have been tested for cytotoxic activity (Table 5). Interestingly, unlike the quantitative studies, which were carried out only on the roots, the cellular studies concerned extracts from shoots and aerial parts. Only one study used animal cells (a mouse breast cancer line), while the majority of the experiments were performed on human cells. When comparing the reported results, a higher sensitivity of mouse cells than human cells to the effect of the tested plant extracts was noted. None of the authors associated cytotoxic properties with the isoflavone content. Moreover, most of the studies did not perform a detailed analysis of the isoflavone content.

#### 3.8.2. In Vivo Studies

The sole animal study, conducted on a mouse model using methanol extract from *O. hirta*, showed an inhibitory effect on the development of breast cancer. In three therapeutic approaches, the effects of *O. hirta* extract, a bacterial strain of *Bifidobacterium longum* subsp. infantis and their combination were tested. A better effect of the extract combined with the bacterial strain was observed, where tumor regression in mice (n = 10) was as much as 30%, while a weaker effect was noted for the extract alone. The average tumor size also differed significantly: 15.60 ± 2.32, 22.50 ± 1.08 and 2.66 ± 0.25 mm^3^ for the extract group, control group and combined group, respectively [66]. As far as the anti-inflammatory properties of *Ononis* are concerned, a sole study described the effect of *O. spinosa* extract and a purified isoflavone fraction (Table 6) [60]. Öz et al. reported that the purified fraction Fr-E5 (containing, among others, ononin and ononin-7-O-glucoside) and ethyl acetate extracts from *Ononis spinosa* were the most effective in reducing induced swelling of the paw (by 32.2%) and ear (by 17.1%), but these results were worse than those of indomethacin used as the reference substance (by 37.9% and 49.7%, respectively). The authors obtained similar results when examining the effect of the extract on the increase in capillary permeability induced by acetic acid. The Fr-E5 fraction counteracted the effect caused by acetic acid, causing a decrease in permeability by 40.3%; this was compared to indomethacin, where the decrease was 49.3% [68].

#### 3.8.3. Antioxidant Potential

Only two representatives of the *Ononis* genus were tested for their antioxidant potential, namely *O. spinosa* and *O. angustissima* (Table 7). In the case of *O. angustissima*, some differences in activity were observed due to the use of different solvents, including butanol, ethyl acetate and dichloromethane. The butanol extract had significantly weaker antioxidant properties [61].

### 3.9. Trifolium sp.

The genus *Trifolium* is one of the largest in the Fabaceae family. It includes almost 300 plant species with many varieties and subspecies [70]. It occurs throughout Europe, especially in the Mediterranean, as well as in Eurasia, North America and East Africa. In total, 125 species have been identified as plants with an ability to assimilate atmospheric nitrogen, which play an important role in agriculture [71]. Most quantitative analyses, including optimizations of the extraction process, were carried out on *T. pratense*. The contents of individual isoflavones depended on the extraction method and conditions. Gligor et al. reported the highest contents of ononin and biochanin A for 60 min extraction using Soxhlet apparatus (6806.60 ± 1020.99 and 102.78 ± 11.31 ng/mL of extract, respectively). However, for formononetin, ultrasonic extraction (30 min, 50 °C) turned out to be more efficient, obtaining 760.05 ± 68.40 ng/mL of the extract [72]. In another study, the most optimal conditions for obtaining daidzein and genistein were ultrasonic extraction (thermal hydrolysis, 50% ethanol) for 10 min (393.23 ± 19.66 and 171.57 ± 8.58 µg/g d.w., respectively) and 30 min (415.07 ± 20.75 and 150.57 ± 7.53 µg/g d.w.). Heat-reflux extraction (50% ethanol, 60 min, without hydrolysis) was equally optimal, with values of 432.30 ± 21.61 daidzein and 154.50 ± 7.72 µg/g d.w. genistein [73]. Rumball et al. noted a change in the contents of isoflavones in *T. repens* depending on the harvest date. Clover herb from cultivar HF1 collected in December contained 1.20 (%*w*/*w* on air-dried material), while this herb collected in January contained 1.95 (%*w*/*w* on air-dried material) [74]. The authors also pointed out notable changes in isoflavone contents based on the morphotic part of the plant. The daidzein contents in the leaves, stems and flowers of *T. glomeratum* were 0.016 ± 0.001, 0.008 ± 0.001 and 0.589 ± 0.015 mg/g d.m., respectively, while the amounts of formononetin were 0.042 ± 0.002, 0.112 ± 0.007 and 0.005 ± 0.001 mg/g d.m. [75].

#### 3.9.1. Cytotoxic Activity

The vast majority of in vitro studies using clover extracts were carried out on breast cancer cell lines (Table 8). One of them described the cytotoxic activity of the extract of *T. pratense* in relation to the extraction method. The IC_50_ for T47D-KBluc breast cancer cells was lower for the extract obtained using the Soxhlet (534 ng/mL) compared to the ultrasonic (477 ng/mL) method, after 24 h of incubation. After 48 h, the difference in effectiveness decreased, but the extract obtained using the Soxhlet method still had the highest score [72]. Zgórka noted that 1000 µg/mL of *T. pratense* extract was the most effective for MCF-7 and MDA-MB-231 cell lines, both after 24 h and 48 h [76]. To increase the penetration of active compounds from the clover extract into the cells, an attempt was made to solubilize the extract. Compared to red clover extract, NeoSolTMRCL40 (product of active solubilization) containing the same amount of isoflavone aglycones showed 3.4 times greater estrogenicity in the MCF-7 cells. The EC_50_ value was 0.361 (dilution factor × 1000) for NeoSolTMRCL40 500 mg vs. 1.237 (dilution factor × 1000) for 100 mg red clover extract. Both preparations increased the percentage of cells in the S phase in a concentration-dependent manner [77]. Very interesting results were obtained from a study on the influence of isoflavones on the molecular metabolism of estrogens in normal and cancer breast cells. It was noted that in nonmalignant ER-negative breast epithelial cells (MCF-10A), the administration of red clover extract had no effect on estrogen metabolic pathways. However, completely different results were obtained on the MCF-7 (ER-positive) malignant tumor cell line. The extract increased the expression of cytochrome CYP 450 1B1, which causes the metabolism of estrogens into 4-hydroxylated catechols; these are oxidized in the next stage to toxic quinones, promoting breast cancer. The expression of CYP 1A, which metabolizes estrogens into non-toxic 2-hydroxylated catechols, was also inhibited [78]. Booth et al. found an inconclusive effect of red clover extract on MCF-7 breast cancer cells. Inhibition of cell growth was observed in only two variants of the tested extracts. However, in the case of the Ishikawa endometrial cell line, it had an effective estrogenic effect [79].

#### 3.9.2. In Vivo Studies

Surprisingly little research using clover extracts has been conducted on animals to determine the chemopreventive potential. The only study determining the anti-inflammatory properties was performed on rats (n = 15) using *T. riograndense* extract at a dose of 100 mg/kg b.w. Carrageenan-induced paw swelling was reduced by 46.9% after 3 h and 65.7% after 4 h following the application of the extract. However, these values were lower than those obtained with indomethacin at a dose of 10 mg/kg b.w.—66.2% and 71.6%, respectively [80].

#### 3.9.3. Antioxidant Potential

*Trifolium pratense*, *T. medium*, *T. alexandrinum*, *T. subterraneum*, *T. thalii* and *T. longidentatum* were tested for their antioxidant activity (Table 9). Gościniak et al. noted that *T. pratense* leaf extracts had lower activity than flower extracts, but both were weaker than the reference ascorbic acid. The exception was the variety Lemmon in the CUPRAC test, where the leaf extract showed greater antioxidant potential [81].

### 3.10. Trigonella sp.

The genus *Trigonella* occurs in Europe, Asia, southern Africa and Australia. It includes about 80 species, the most popular of which is *Trigonella foenum-graecum* Linn., used for medicinal, cosmetic and food purposes [85]. The isoflavones in the *Trigonella* species are limited to only two: genistein and daidzein in the leaves and stems. The content of genistein in *Trigonella* stems was notably higher (66.3 mg/kg d.m.) than the amount determined in the leaves (9.1 mg/kg d.m.). The contents of daidzein in the leaves varied between 18.1 and 23.1 mg/kg d.m., while there was no quantitative determination in the stems. However, only one author has carried out quantitative and qualitative isoflavones analyses [44].

#### 3.10.1. Cytotoxic Activity

In vitro studies using extracts from *Trigonella* sp. have tried to explain the molecular basis for its impact on MCF-7 breast cancer cells. Salama et al. [86] assessed the biological activity of various raw materials from *T. foenum-graecum* Linn. (air-dried leaves, untreated seeds, germinated seeds, soaked seeds, boiled seeds), noting that in all biological tests, the most effective was the extract from air-dried seeds at a previously determined IC_50_ dose in relation to the tested cell line. The extract enhanced apoptosis processes by increasing the transcription of p53 protein, caspase-3 and BAX protein (2.15, 3.76 and 2.88 of fold change vs. untreated control, respectively). All these proteins are responsible for intensifying apoptosis or repairing damaged DNA. It is worth noting, however, that the results obtained were lower than those for the reference, doxorubicin (3.93, 5.03 and 3.23 of fold change, respectively). In turn, the decrease in the value of BCL-2 protein (0.35 of fold change), whose function is to inhibit apoptosis, was greater than in the group treated with doxorubicin (0.12 of fold change). A significant decrease in 8-hydroxy-2′-deoxyguanosine after the use of air-dried seed extract (604 pg/mg) suggested the inhibition of oxidative processes causing changes in RNA. This study indicated a multidirectional effect of the *Trigonella* extract on MCF-7 cells by stimulating the production of factors that enhance apoptosis and reducing the expression of apoptosis-inhibiting protein. Additionally, the antioxidant potential was noted, resulting from the reduced amount of hydroxy-2′-deoxyguanosine [86].

Khoja et al. [87] observed that the number of extractions performed may affect the cytotoxic activity against cancer cells. The IC_50_ value for the extract from *T. foenum-graecum* L. seeds after triple extraction (812 μg/mL) was much lower than after single extraction (5605 μg/mL). A notable difference between the two extracts was also observed in cell viability after 48 and 72 h of incubation, with 47.3% and 33.52% in the single extraction group and 25.4% and 13.8% in the triple extraction group, respectively. The extract after triple extraction showed significantly promoted cell mtDNA damage, but the results were worse than those obtained when using hydrogen peroxide as the positive control [87].

#### 3.10.2. Antioxidant Potential

The only study available in the literature on the antioxidant properties of the extract provided data from a DPPH test. The percentage of inhibition for *T. isthmocarpa* for methanolic seed extract was 78.2%, for methanolic area parts’ extract was 87.2% and for BHT (reference substance) was 73.1% [88].

### 3.11. Vicia sp.

The genus Vicia includes 166 species of annual and perennial plants. It is widespread in Europe, Asia, America and the temperate regions of South America and tropical Africa [89]. The most well-known species is *Vicia faba* L. (broad bean), cultivated for food. Research on the Vicia genus has so far focused on the quantitative and qualitative determination of the contents of isoflavones in various parts of the plant. Jaihyunk et al. determined the contents of isoflavones in several genotypes of the *Vicia faba* species. The morphotic parts analyzed were leaves, immature pods and grains. The authors determined the highest maximum values of daidzin and genistin in the leaves, and daidzein and genistein in the grains [90]. Kaufman et al. determined high contents of daidzein and genistein in one of the examined specimens of *Vicia faba* stems, where the values reached 1032.8 and 92.1 mg/kg d.m., respectively [44].

#### Antioxidant Potential

Only one study examining the antioxidant potential was performed on *Vicia faba*. In the DPPH test, the IC_50_ values obtained by for extract (20.7 μg/mL) were higher than the reference substances used (8.28 ± 0.50 μg/mL for BHT vs. 12.4 ± 1.1 μg/mL for BHA). However, the results of the reducing power assay were surprising. Despite a weaker reducing effect compared to BHT, the extract was much more effective than BHA (32.6 ± 2.5 vs. 1057 ± 78 (mg AAE/g d.w.)) [91].

### 3.12. Other Species

Despite the numerous data for different *Fabaceae* species, some of them have so far barely been examined. These include *Coronilla*, *Lotus* and *Pisum* species, for which only scarce data exist on their isoflavone contents, while no information was found on their chemopreventive potential. The genus *Coronilla* includes 24 species, naturally occurring throughout Europe (introduced in Scandinavia and the British Isles), Africa and western Asia. Information on the quantified contents in the genus *Coronilla* sp. is limited to one study, which reported low contents of genistin that were similar before and after hydrolysis (1.10 and 1.11 μg/mL, respectively) [23]. The *Lotus* genus includes 125 species, naturally distributed all over the world, except the Far East of Russia and Southeast Asia. The only species of the genus *Lotus* sp. tested for its isoflavone content was *Lotus corniculatus*. Quantitative analysis showed the presence of only two isoflavones before (genistin, genistein) and three after (genistin, genistein, daidzin) acid hydrolysis. Genistin showed the highest content of all detected compounds, and that increased from 0.75 to 2.13 μg/mL after hydrolysis [23]. In the case of peas, there is a large variability in the contents of isoflavones. In the relevant study, 100 specimens were prepared, differing in morphological features, such as the colors of flowers and seeds, as well as countries of origin. The total isoflavone contents varied significantly. For example, in a specimen from Ethiopia, 94.71 ng/g d.w. was reported, and in a specimen from Afghanistan, it was as much as 25.402 ng/g d.w. [92]. However, there are no in vitro and in vivo studies on chemoprevention against hormone-dependent cancer cells.

## 4. Discussion

The well-known associations between isoflavones and the prevention of hormone-dependent cancers inspired us to take a closer look at the Fabaceae plants, containing these phytoestrogens, to estimate their chemopreventive potential and preselect the species that are interesting for future studies. Apart from the most-examined species like soy and clover, which are supportively used in some hormonal disorders, most of the species of the family are rather poorly investigated in terms of their biological activity. Surprisingly, for some species (e.g., *Medicago*, *Trifolium*), there are lots of reports on their isoflavone contents, which, in some cases, are really satisfactory, but still the data on their impact on hormone-dependent cancer cells are scarce. The existing information is usually limited to the determination of the impact on cell viability, while other aspects are omitted. Moreover, there is a similar situation in the anti-inflammatory potential of Fabaceae species, with only limited data, usually from in vitro studies. The most intensively studied aspect of the chemoprevention of Fabaceae species is their antioxidant potential, and the interesting results on this justify further studies.

Numerous limitations and shortcomings of the studies included in this review make it difficult to assess the chemopreventive properties of plants from the Fabaceae family containing isoflavones. One of the most important limitations is the issue of quantification of the compounds of interest. While authors usually defined extracts well in terms of qualitative phytochemical contents, quantitative studies were sporadic. In some studies, only the total content of flavonoids was provided, without distinguishing isoflavones or individual substances. Only a few publications provided information on the potential relationship or correlation between the chemopreventive effect and the content of isoflavones or other groups of compounds. However, some studies proved the superiority of an infusions over organic extracts in terms of its effectiveness on cancer cells, which suggests that the activity did not result from isoflavones’ presence, as the group of compounds is poorly soluble in water. Another difficulty in synthesizing the research results arises from a lack of general standardization in providing data, with different concentration units and information routinely published (e.g., whether the given value refers to the mass of the extract or the dry or fresh mass of plants), which make it difficult to compare the results.

In vitro studies mainly referred to the cytotoxicity and viability of cancer cells, while no studies were conducted to determine cell migration or proliferation, though this information is crucial for the elimination of cancer cells. Similarly, there were few advanced studies determining the mechanisms of action of extracts on the regulation of the pathway of apoptosis and the process of stopping the cell cycle. This aspect should be explored in the future. Surprisingly often, tests were carried out without a reference substance, though including one not only provides information about the effectiveness of the tested extracts but also ensures that the experiment is carried out correctly. Similarly, some studies of the antioxidant potential did not have a standard substance, which makes it near-impossible to use their results. Last, typically, experiments lasting 24 or 48 h were performed, while there were no studies determining the long-term effect of the substance on cancer cells.

In vivo studies were performed only rarely and were focused on the anti-inflammatory effect. The studies were well-planned, with control groups, including the use of a reference drug, but in some cases, the study groups were too small to draw definite conclusions. Moreover, most of the studies were only descriptive, with no mechanistic aspects explained.

At the same time, it is worth mentioning the positive aspects of the research conducted. The research covers a very large number of species from the Fabaceae family, and in the vast majority of analyses, not only was the qualitative but also the quantitative content of individual isoflavones determined. Moreover, the approaches to extraction methods, the type of solvent used and the part of the plant subjected to analysis within the same genus were very diverse. The standard seems to be conducting studies of the antioxidant potential, probably due to the ease of such analyses.

## 5. Conclusions

To conclude, in our opinion, despite the reported weak or moderate activity of the reviewed Fabaceae species, they still have the potential to be used in hormone-dependent cancer chemoprevention. This is particularly true for *Ononis* species of plants, which have demonstrated interesting anticancer and anti-inflammatory properties in animal studies, if we can overcome their potent cytotoxic and antioxidant activity demonstrated in vitro. Moreover, plants from *Genista* and *Trifolium* species are in our opinion promising and should be studied more intensively. However, there is a lot of progress still to be made, especially in the (i) optimization of the extraction conditions, to obtain isoflavone-rich extracts; (ii) analysis of the relationships between the isoflavone content of the species and the observed activity; (iii) possible enhancement in the activity between Fabaceae plants and the cytostatic used in the treatment of hormone-dependent cancers; (iv) determination of the safety profiles of the tested species; and (v) the formulation of extracts or fractions from the most promising species.

## Figures and Tables

**Table 1:** Antioxidant potential of *Cytisus* sp.

Extract	Effects	References
*Cytisus multiflorus* (flowers)	Oxidative hemolysis inhibition assay: IC_50_ no activity for EtOH extract vs. 109 ± 9 (µg/mL) for infusion vs. 85 ± 2 for Trolox. Thiobarbituric acid reactive substance assay: IC_50_ 3.7 ± 0.1 (µg/mL) for EtOH extract vs. 5.3 ± 0.1 for infusion vs. 23 ± 2 for Trolox.	[27]
*Cytisus villosus * (aerial parts)	DPPH: IC_50_ 59 ± 2 (μg/mL) for H_2_O extract vs. 31 ± 2 for EtOAc extract vs. 3.1 ± 0.1 for ascorbic acid. ABTS: IC_50_ 468 ± 34 (μg/mL) for H_2_O extract vs. 232 ± 2 for EtOAc extract vs. 101 ± 3 for ascorbic acid.	[26]
*Cytisus villosus* Pourr. (leaves)	DPPH: IC_50_ 3.94 ± 0.09 (μg/mL) for H_2_O extract vs. 4.81 ± 0.061 for EtOH extract vs. 4.15 ± 0.19 for BHT. ABTS: IC_50_ 2.88 ± 0.07 (μg/mL) for H_2_O extract vs. 3.32 ± 0.12 for EtOH extract vs. 2.14 ± 0.07 for ascorbic acid. RP: IC_50_ 1.94 ± 0.10 (μg/mL) for H_2_O extract vs. 2.69 ± 0.06 for EtOH extract.	[28]

EtOAc ethyl acetate, EtOH ethanol, H_2_O water extracts.

**Table 2:** Cytotoxic and anti-inflammatory potential of *Genista* sp.—in vitro studies.

Species	Experiment	Effects	References
cytotoxic properties			
*Genista acanthoclada*, *Genista hassertiana*, *Genista depressa* and *Genista millii * (aerial parts)	Cell lines: MCF-7 and Ishikawa Extract: EtOAc and MeOH at concentrations of 0.01, 0.1 and 1 μg/mL on Ishikawa cells and 1 μg/mL on MCF-7 cells Reference: estradiol (0.1 nM) Different groups: none Methods: estrogen agonist activity	no estrogen-antagonist activity in tested concentrations. most effective agonist at a dose of 1 μg/mL MeOH extract of *G. hassertiana*—100.7 ± 9.8 (% of estradiol) in Ishikawa cells. most effective agonist at a dose of 1 μg/mL MeOH extract of *G. millii*—115.5 ± 16.6 (% of estradiol) in MCF-7 cell group.	[33]
*Genista sessilifolia* (aerial parts)	Cell lines: MCF-7, MDA-MB-231, HeLa, LNCaP Extract: MeOH ar concentrations of 0.5, 0.75 and 1.5 mg/mL, for 24 and 48 h Reference: none Different groups: untreated cells Methods: morphological analysis, Western blot	all cell lines were susceptible to the extract. MDA-MB-231 cell line was more sensitive than others. ↓ cell cycle—↑ p21, Rb. ↑ apoptosis—↑ BAD, TRAIL, p53, caspases 8, 9, 3/7.	[34]
*Genista tridentata* (flowers)	Cell lines: MCF-7, HeLa Extract: hydroethanolic and infusion (6.25–400 μg/mL) Reference: ellipticine (0.91–3.2 µg/mL) Different groups: untreated cells Methods: sulforhodamine B assay	GI_50_: 146.8 ± 6.5 (µg/mL) for hydroethanolic extract vs. 129.1 ± 6.3 for infusion vs. 0.91 ± 0.04 for ellipticine in MCF-7 cells. GI_50_: 102.9 ± 10.6 (µg/mL) for hydroethanolic extract vs. 83.2 ± 6.5 for infusion vs. 1.91 ± 0.06 for ellipticine in HeLa cells.	[28]
*Genista monspessulana* (seeds)	Cell lines: PC-3, SiHa Extract: EtOH (0.8–500 µg/mL) Reference: curcumin (0.16–100 µg/mL) Different groups: untreated cells Methods: MTT assay	average IC_50_: 98.3 ± 68.1 µg/mL in PC-3 cells and 148.8 ± 97.8 µg/mL in SiHa cells. more effective extracts for seeds collected in woodland space.	[35]
anti-inflammatory properties			
*Genista tridentata* (stems, leaves and roots)	Cell line: macrophages RAW 264.7 stimulated by LPS Extract: EtOH at a concentration of 100 μg/mL Reference: none Different groups: none Methods: biochemical analysis, Western blot	↓ mRNA IL-1b, mRNA COX-2, COX-2 for stem and root extracts. ↓ NO, mRNA NOS2, iNOS, mRNA IL-6 for all extracts. ↑ TNF-α for all extracts.	[36]
*Genista tridentata* (flowers)	Cell line: macrophages RAW 264.7 stimulated by LPS Extract: hydroethanolic extract and infusion (6.25–400 μg/mL) Reference: dexamethasone Different groups: none Methods: biochemical analysis NO	IC_50_ 207.4 ± 15.5 (µg/mL) for hydroethanolic extract vs. 144.4 ± 2.2 for infusion vs. 6 ± 1 for dexamethasone.	[27]

Cell lines: MCF-7 (human breast adenocarcinoma), SiHa (human cervix squamous carcinoma), MDA-MB-231 (human breast adenocarcinoma), HeLa (human cervix adenocarcinoma), Ishikawa (human uterus cancer). Extracts: EtOAc (ethyl acetate), MeOH (methanol), EtOH (ethanol), ↑ increase, ↓ decrease.

**Table 3:** Antioxidant potential of *Genista* sp.

Extract	Effects	References
*Genista aspalathoides* Lamk ssp. *aspalathoides* (aerial parts)	DPPH: IC_50_ 14.49 ± 0.94 (μg/mL) for n-BuOH extract vs. 6.87 ± 0.15 for Trolox vs. 2.19 ± 0.26 for ascorbic acid.	[39]
*Genista cadasonensis* Valsecchi (aerial parts)	DPPH: IC_50_ 10.36 ± 0.84 (mg/mL) for DCM extract vs. 2.98 ± 0.81 for Ace extract vs. 2.42 ± 0.63 for MeOH extract vs. 0.18 ± 0.32 for BHA. ABTS: IC_50_ 2.83 (mg/mL) for DE vs. 0.88 ± 0.62 for (AE) vs. 1.1 ± 0.73 for MeOH vs. 0.17 for BHA.	[40]
*Genista sandrasica* (aerial parts)	DPPH: inhibition % 15.90 ± 0.42, 24.77 ± 3.43 and 46.16 ± 1.09 for extracts before acid hydrolysis vs. 87.65 ± 0.28, 91.61 ± 0.06 and 92.57 ± 0.10 for gallic acid at concentrations of 0.25, 0.5 and 1 mg/mL, respectively. DPPH: inhibition % 69.51 ± 2.85, 85.64 ± 0.59 and 87.18 ± 0.08 for extracts after acid hydrolysis vs. 87.65 ± 0.28, 91.61 ± 0.06 and 92.57 ± 0.10 for gallic acid at concentrations of 0.25, 0.5 and 1 mg/mL, respectively. Ferrous ion-chelating capacity: inhibition % 6.89 ± 0.88, 8.29 ± 0.71 and 8.21 ± 0.42 for extracts before acid hydrolysis vs. n.d., 21.71 ± 1.10 and 26.94 ± 1.48 for butylated hydroxyanisol at concentrations of 0.25, 0.5 and 1 mg/mL, respectively.	[38]
*Genista tenera* (aerial parts)	DPPH: bleaching percent (139.1 µg/mL) 39.1 (%) for diethyl ether extract vs. 48.7 for EtOAc extract vs. 24 for n-BuOH extract vs. 96 for quercetin.	[41]
*Genista tridentata* (flowers)	Oxidative hemolysis inhibition assay: IC_50_ 76 ± 5 (µg/mL) for EtOH extract vs. 78 ± 6 for infusion (I) vs. 85 ± 2 for Trolox. Thiobarbituric acid reactive substance assay: IC_50_ 3.19 ± 0.02 (µg/mL) for EtOH vs. 5.3 ± 0.1 for I vs. 23 ± 2 for Trolox.	[27]
*Genista vuralii* (aerial parts)	DPPH: inhibition % 16.85 ± 1.25, 29.91 ± 0.01 and 50.70 ± 3.87 for extracts before acid hydrolysis vs. 87.65 ± 0.28, 91.61 ± 0.06 and 92.57 ± 0.10 for gallic acid at concentrations of 0.25, 0.5 and 1 mg/mL, respectively. DPPH: inhibition % 69.80 ± 3.27, 85.05 ± 0.25 and 86.12 ± 0.42 for extracts after acid hydrolysis vs. 87.65 ± 0.28, 91.61 ± 0.06 and 92.57 ± 0.10 for gallic acid at concentrations of 0.25, 0.5 and 1 mg/mL, respectively. Ferrous ion-chelating capacity: inhibition % 3.28 ± 1.24, 7.47 ± 0.11 and 10.21 ± 1.7 for extracts before acid hydrolysis vs. n.d., 21.71 ± 1.10 and 26.94 ± 1.48 for butylated hydroxyanisol at concentrations of 0.25, 0.5 and 1 mg/mL, respectively. Ferrous ion-chelating capacity: inhibition % 8.83 ± 0.89, 8.95 ± 0.65 and 13.90 ± 0.91 for extracts after acid hydrolysis vs. n.d., 21.71 ± 1.10 and 26.94 ± 1.48 for butylated hydroxyanisol at concentrations of 0.25, 0.5 and 1 mg/mL, respectively.	[38]

Ace acetone, EtOAc ethyl acetate, EtOH ethanol, MeOH methanol, n-BuOH n-butanol extracts.

**Table 4:** Antioxidant potential of *Lupinus* sp.

Extract	Effects	References
*Lupinus albus* L. (hypocotyl)	DPPH: IC_50_ 202 ± 6 (µM Trolox equivalents/100 g fresh weight) for methanolic hypocotyl extract.	[46]
*Lupinus angustifolius* L. (hypocotyl)	DPPH: IC_50_ 250 ± 10 (µM Trolox equivalents/100 g fresh weight) for methanolic hypocotyl extract.	[46]
*Lupinus mutabilis Sweet* (seeds)	DPPH: IC_50_ 720 ± 20 (µM Trolox equivalents/100 g fresh weight) for methanolic seed coat extract.	[46]
*Lupinus angustifolius* L. (seeds)	DPPH: IC_50_ 19.1 ± 0.2 (mg Trolox/g sample) for methanolic seed extract 9 days after germination.	[48]
*Lupinus mutabilis* L. (seeds)	DPPH: IC_50_ 63.2 (µM Troxol (TE) per 100 g dry seed powder) for ethanolic ecotype extract grown on silty loam soil and extracted with citric acid.	[49]

**Table 5:** Cytotoxic potential of *Ononis* sp.—in vitro and in vivo studies.

Species	Experiment	Effects	References
in vitro studies			
*Ononis spinosa* L. (aerial parts)	Cell lines: MCF-7 and SiHa. Extract: MeOH. Concentration range: 50–250 μg/mL. Reference: none. Different groups: control (DMSO). Methods: crystal violet assay.	MCF-7 IC_50_: 101.28 ± 8.29 μg/mL. SiHa IC_50_: 181.96 ± 28.37 μg/mL.	[64]
*Ononis spinosa* L. (shoots)	Cell line: MDA-MB-231. Extract: EtAOc. Concentration range: 10–100 μg/mL. Reference: tamoxifen. Different groups: control (DMSO). Methods: cytotoxicity assay.	Dose-dependent effect—most effective dose 100 μg/mL. ↓ 65 ± 0.4% growth of cells at 100 μg/mL. IC_50_: 28.75 ± 2.5 μg/mL for ethyl acetate extract vs. 10.5 ± 1.7 μg/mL for tamoxifen.	[65]
*Ononis hirta* L. (aerial parts)	Cell line: 66 cl-4-GFP. Extracts: EtOH, CHCl_3_, H_2_O, n-Hex, MeOH, BuOH. Concentration range: 4.56–13.73%. Reference: vincristine sulfate 0.05–100 nM. Different groups: control (DMSO), untreated control. Methods: MTT assay.	The highest score—methanolic and chloroform extracts. IC_50_: 16.66 ± 1.77 μg/mL for methanolic extract vs. 45 nM for vincristine sulfate.	[66]
*Ononis hirta* L. and *Ononis siculata* L. (aerial parts)	Cell line: MCF-7. Extracts: H_2_O, n-Hex, MeOH, BuOH. Concentrations range: 5–200 μg/mL. Reference: vincristine sulfate (0.05–100 nM). Different groups: control (DMSO), untreated control. Methods: MTT assay.	*Ononis hirta* IC_50_: 27.96 ± 0.54 μg/mL for methanolic extract vs. 44.58 ± 1.42 for chloroform extract vs. 72.06 ± 2.79 for n-hexane extract. *Ononis siculata* IC_50_: 66.02 ± 1.58 μg/mL for chloroform extract vs. 114.11 ± 2.42 for n-hexane extract.	[67]
*Ononis natrix* L. (aerial parts)	Cell line: MCF-7. Extract: EtOH. Concentrations range: 0.1–100 μg/mL. Reference: vincristine sulfate (0.05–100 nM). Different groups: none. Methods: Trypan blue assay.	IC_50_: 90.01 ± 4.68 μg/mL.	[22]
in vivo studies			
*Ononis hirta* (aerial parts)	Animal model: breast-tumor-bearing female mice Balb/C mice (n = 10). Extract: methanolic: 28.5 mg/mL intraperitoneally (i.p.) used daily for 14 days, 9 days after tumor cells’ inoculation. Different groups: control (5% Tween 20 w PBS), intratumoral injection (100 μL, 1.5 × 10^7^ bacterial cells) of *Bifidobacterium longum*, combined group. Methods: tumor size measured every 2 days for 14 days. Histological assessment.	Final tumor size: 15.60 ± 2.32 (mm^3^) for extract group vs. 22.50 ± 1.08 for control group vs. 2.66 ± 0.25 for combined group. Change % in body weight: −1.61 for extract group vs. 14.57 for control group vs. −2.97 for combined group.	[66]

Cell line: MCF-7 (human breast adenocarcinoma), SiHa (human cervix squamous carcinoma), MDA-MB-231 (human breast adenocarcinoma), 66 cl-4-GFP (mouse mammary gland); CHCl_3_ chloroform, EtOAc ethyl acetate, EtOH ethanol, H_2_O water, n-Hex n-hexane, MeOH methanol, n-BuOH n-butanol extracts, ↓ decrease.

**Table 6:** Anti-inflammatory potential of *Ononis* sp.—in vivo studies.

Species	Experiment	Effects	References
*Ononis spinosa* L. subsp. *Liosperma* (roots)	Experiment 1 (E1): Animal model: carrageenan-induced paw edema in male Swiss albino mice and Sprague–Dawley rats (n = 18). Extract: EtOAc (6 fractions: Fr-E1–Fr-E6) 100 mg/kg orally administered 60 min before carrageenan injection. Reference: indometacin 10 mg/kg b.w. Different groups: negative control. Methods: paw volume measured at 1.5, 3, 4.5 and 6 h after injection. Experiment 2 (E2): Animal model: acetic-acid-induced increase in capillary permeability in male Swiss albino mice and Sprague–Dawley rats (n = 18) Extract: EtOAc (6 fractions: Fr-E1–Fr-E6) 100 mg/kg orally administered 40 min before acetic acid (i.p.) injection. Reference: indomethacin 10 mg/kg b.w. Different groups: negative control. Methods: spectrophotometric measure of Evans blue concentration (i.v. injection 10 min before acetic acid). Experiment 3 (E3): Animal model: TPA-induced male Swiss albino mice and Sprague–Dawley rats with ear edema (n = 18). Extract: EtOAc (6 fractions: Fr-E1–Fr-E6) 0.5 mg/ear on surface of ear after TPA application. Reference: indomethacin 0.5 mg/ear. Different groups: negative control. Methods: weight edema and swelling thickness measured 4 h after applications.	E1: ↓ paw volume inhibition by 28.1% and 32.2% in Fr-E5 group vs. 28.1% and 37.9% in indomethacin group, after 4.5 and 6 h, respectively. E2: ↓ Evans blue concentration inhibition by 40.3% in Fr-E5 group vs. 49.3% in indomethacin group. E3: ↓ weight edema inhibition by 22.5% in Fr-E5 group vs. 69.6% in indomethacin group. E3: ↓ swelling thickness inhibition by 17.1% in Fr-E5 group vs. 49.7% in indomethacin group.	[60]

↓ decrease.

**Table 7:** Antioxidant potential of *Ononis* sp.

Extract	Effects	References
*Ononis angustissima* L. (roots)	DPPH: IC_50_ 66.87 ± 1.97 (μg/mL) for CHCl_3_ extract vs. 24.48 ± 0.55 for EtOAc extract vs. 189.08 ± 1.99 for BuOH extract vs. 4.89 ± 0.18 for quercetin vs. 9.23 ± 0.38 for BHT. ABTS: IC_50_ 56.08 ± 0.63 (μg/mL) for CHCl_3_ extract vs. 49.25 ± 1.18 for EtOAc extract vs. 112.91 ± 1.43 for BuOH extract vs. 6.94 ± 0.73 for quercetin. Fer(III): IC_50_ 112.54 ± 2.04 (μg/mL) for CHCl_3_ extract vs. 63.42 ± 0.78 for EtOAc extract vs. 141.37 ± 2.52 for BuOH extract vs. 41.64 ± 0.83 for BHT.	[69]
*Ononis spinosa* L. (aerial parts)	DPPH: IC_50_ 96 ± 0.97 (mg Trolox (TE) per g extract) for MeOH extract. ABTS: IC_50_ 61.29 ± 0.82 (mg TE per g extract) for MeOH extract. Phosphomolybdenum method: 1.50 ± 0.11 (mmol TE per g extract) for MeOH extract. Cupric-ion-reducing activity: 101.44 ± 1.05 (mg TE per g extract) for MeOH extract. Ferric-reducing antioxidant power: 59.61 ± 0.87 (mg TE per g extract) for MeOH extract. Metal-chelating activity: 7.55 ± 0.72 (mg EDTAE per g extract) for MeOH extract.	[56]
*Ononis spinosa* L. (roots)	DPPH: IC_50_ 48.17 (μg/mL) for EtOAc extract (Fr-E5) vs. 4.21 for ascorbic acid. ABTS: IC_50_ 62.82 (μg/mL) for EtOAc extract (Fr-E5) vs. 8.62 for ascorbic acid. Reducing power assay: ↓ 31.08% for EtOAc extract (Fr-E5) vs. 67.81% for ascorbic acid. OH radical inhibition: IC_50_ 44.36 (μg/mL) for ethyl EtOAc (Fr-E5) vs. 5.57 for ascorbic acid.	[60]

CHCl_3_ chloroform, EtOAc ethyl acetate, MeOH methanol, BuOH butanol extracts. ↓ decrease.

**Table 8:** Cytotoxic potential of *Trifolium* sp.—in vitro studies.

Species	Experiment	Effects	References
*Trifolium pratense* L.	Cell line: T47D-KBluc. Extract: EtOH (50–1000 µg/mL) for 24 or 48 h. Reference: none. Different groups: untreated cells. Methods: Alamar Blue assay.	IC_50_: 534 (519–550) ng/mL for Soxhlet extraction vs. 477 (462–493) ng/mL for ultrasound extraction after 24 h. IC_50_: 332 (323–342) ng/mL for Soxhlet extraction vs. 314 (305–323) ng/mL for ultrasound extraction after 48 h.	[72]
*Trifolium pratense* L.	Cell lines: MCF-7, MDA-MB-231. Extract: EtOH (1–1000 µg/mL) for 24 or 48 h. Reference: none. Different groups: untreated cells. Methods: MTT assay.	dose-dependent effect—most effective dose 100 μg/mL. dose-dependent cytotoxic effect for both times. most effective dose 1000 μg/mL for both cell lines.	[76]
*Trifolium pratense* L.	Cell line: MCF-7. Extract: EtOH (100 mg), NeoSolTM ^TM^RCL 500 (500 mg, 20%), NeoSolTM ^TM^RCL 250 (250 mg, 20%), NeoSolTM ^TM^RCL 100 (100 mg, 20%). Reference: none. Different groups: none. Methods: estrogenic assay.	lowest EC_50_ (% of the effect of 17β-estradiol 0.1 nM) in NeoSolTM ^TM^RCL 500 group (0.361 (dilution factor × 1000)) vs. 1.237 in Trifolium extract group.	[77]
*Trifolium pratense* L.	Cell lines: MCF-7, MCF-10A. Extract: *T. pratense* (30% of isoflavones—biochanin A (14.47%), formononetin (14.26%), genistein (0.41%) and daidzein (0.23%)) at a dose of 10 µg/mL. Reference: none. Different groups: none. Methods: LC-MS/MS assay of estrogen metabolism, RT-qPCR assay of gene expression.	no effect of *Trifolium* extract on 2-MeOE1 and 4-MeOE1 levels in normal breast cells in MCF-10A cell lines. ↑ expression of CYP 450 1B1 and ↑ genotoxicity of 4-MeOE1 in MCF-7 cell lines.	[78]
*Trifolium pratense* L.	Cell lines: Ishikawa, MCF-7. Extract: 16 EtOH extracts from aboveground parts and flower heads in dose of 20 µg/mL. Reference: tamoxifen citrate (10 and 30 μM). Different groups: negative control (0.25% DMSO), positive control (1 μM 17β-estradiol). Methods: XTT assay (MCF-7), estrogenic assay (Ishikawa).	alkaline phosphatase induction > 50% for extracts from aboveground parts, <50% for extracts from flower heads in estrogenic assay in Ishikawa cells. inhibition of MCF-7 cell growth only using 2 extracts in the XTT assay.	[79]

↑ increase.

**Table 9:** Antioxidant potential of *Trifolium* sp.

Extract	Effects	References
*Trifolium pratense* L. var. Tenia (leaves and flowers)	DPPH: IC_50_ 6.352 ± 0.262 (mg/mL) for ethanolic leaf extracts vs. 3.033 ± 0.129 (mg/mL) for ethanolic flower extracts vs. 0.068 ± 0.005 for ascorbic acid CUPRAC: IC_50_ 2.049 ± 0.108 (mg/mL) for ethanolic leaf extracts vs. 1.206 ± 0.012 for ethanolic flower extracts vs. 0.073 ± 0.003 for ascorbic acid	[81]
*Trifolium pratense* L. var. Atlantis (leaves and flowers)	DPPH: IC_50_ 6.469 ± 0.106 (mg/mL) for ethanolic leaf extracts vs. 2.280 ± 0.039 (mg/mL) for ethanolic flower extracts vs. 0.068 ± 0.005 for ascorbic acid CUPRAC: IC_50_ 1.856 ± 0.062 (mg/mL) for ethanolic leaf extracts vs. 0.814 ± 0.0012 for ethanolic flower extracts vs. 0.073 ± 0.003 for ascorbic acid	[81]
*Trifolium pratense* L. var. Lucrum (leaves and flowers)	DPPH: IC_50_ 4.854 ± 0.273 (mg/mL) for ethanolic leaf extracts vs. 1.393 ± 0.096 (mg/mL) for ethanolic flower extracts vs. 0.068 ± 0.005 for ascorbic acid CUPRAC: IC_50_ 1.710 ± 0.029 (mg/mL) for ethanolic leaf extracts vs. 0.644 ± 0.004 for ethanolic flower extracts vs. 0.073 ± 0.003 for ascorbic acid	[81]
*Trifolium pratense* L. var. Magellan (leaves and flowers)	DPPH: IC_50_ 7.924 ± 0.082 (mg/mL) for ethanolic leaf extracts vs. 2.375 ± 0.060 (mg/mL) for ethanolic flower extracts vs. 0.068 ± 0.005 for ascorbic acid CUPRAC: IC_50_ 3.363 ± 0.067 (mg/mL) for ethanolic leaf extracts vs. 0.955 ± 0.019 for ethanolic flower extracts vs. 0.073 ± 0.003 for ascorbic acid	[81]
*Trifolium pratense* L. var. Lemmon (leaves and flowers)	DPPH: IC_50_ 2.583 ± 0.089 (mg/mL) for ethanolic leaf extracts vs. 2.197 ± 0.057 (mg/mL) for ethanolic flower extracts vs. 0.068 ± 0.005 for ascorbic acid CUPRAC: IC_50_ 0.683 ± 0.0037 (mg/mL) for ethanolic leaf extracts vs. 0.832 ± 0.0017 for ethanolic flower extracts vs. 0.073 ± 0.003 for ascorbic acid	[81]
*Trifolium pratense* L. var. Milena (leaves and flowers)	DPPH: IC_50_ 4.090 ± 0.029 (mg/mL) for ethanolic leaf extracts vs. 2.125 ± 0.044 (mg/mL) for ethanolic flower extracts vs. 0.068 ± 0.005 for ascorbic acid CUPRAC: IC_50_ 1.266 ± 0.045 (mg/mL) for ethanolic leaf extracts vs. 0.723 ± 0.029 for ethanolic flower extracts vs. 0.073 ± 0.003 for ascorbic acid	[81]
*Trifolium pratense* L. (flowers)	DPPH: IC_50_ 26.27 ± 0.31 (µg TE/ g d.w.) for ethanolic flower extract ABTS: IC_50_ 638.55 ± 9.14 (µg TE/ g d.w.) for ethanolic flower extract FRAP: IC_50_ 526.86 ± 3.21 (mg FS/g d.w.) for ethanolic flower extract	[73]
*Trifolium pratense* L. (flowers)	DPPH: inhibition (%) 90.4 ± 0.06	[82]
*Trifolium pratense* L. (flowers)	Superoxide Anion Scavenging: 4.07 μmol GAE/100 g freeze-dried mass Peroxyl Radical Scavenging: 88.25 mmol TE/100 g freeze-dried mass Fremy’s Salt Scavenging: 15.40 μmol GAE/100 g freeze-dried mass	[83]
*Trifolium pratense* L. (flowers)	Superoxide Anion Scavenging: 1.92 μmol/100 g freeze-dried mass Peroxyl Radical Scavenging: 130.00 mmol TE/100 g freeze-dried mass Fremy’s Salt Scavenging: 15.40 μmol GAE/100 g freeze-dried mass	[83]
*Trifolium pratense* L. subsp. Nivale (flowers)	Superoxide Anion Scavenging: 2.19 μmol GAE/100 g freeze-dried mass Peroxyl Radical Scavenging: 21.60 mmol TE/100 g freeze-dried mass Fremy’s Salt Scavenging: 9.40 μmol GAE/100 g freeze-dried mass	[83]
*Trifolium pratense* L. subsp. nivale (flowers)	Superoxide Anion Scavenging: 1.58 μmol GAE/100 g freeze-dried mass Peroxyl Radical Scavenging: 110.85 mmol TE/100 g freeze-dried mass Fremy’s Salt Scavenging: 5.54 μmol GAE/100 g freeze-dried mass	[83]
*Trifolium medium* L.	DPPH: IC_50_ 30.18 ± 0.37 (µg/mL) for ethanolic extracts vs. 3.00 ± 0.90 (µg/mL) TE vs. 0.89 ± 1.80 for GAE ABTS: IC_50_ 30.30 ± 0.18 (µg/mL) for ethanolic extracts vs. 2.75 ± 0.07 (µg/mL) for TE vs. 3.00 ± 0.90 for GAE CUPRAC: 48.00 ± 1.87 (mg GAE/g dm) for ethanolic extracts Folin–Ciocalteu: 107.50 ± 0.26 (mg GAE/g dm) for ethanolic extracts	[76]
*Trifolium alexandrinum* (flowers)	Superoxide Anion Scavenging: 2.12 μmol GAE/100 g freeze-dried mass Peroxyl Radical Scavenging: 28.00 TE/100 g freeze-dried mass Fremy’s Salt Scavenging: 5.37 μmol GAE/100 g freeze-dried mass	[83]
*Trifolium subterraneum * (flowers)	Superoxide Anion Scavenging: 1.82 μmol GAE/100 g freeze-dried mass Peroxyl Radical Scavenging: 137.50 mmol TE/100 g freeze-dried mass Fremy’s Salt Scavenging: 1.47 μmol GAE/100 g freeze-dried mass	[83]
*Trifolium subterraneum* (flowers)	Superoxide Anion Scavenging: 0.61 μmol GAE/100 g freeze-dried mass Peroxyl Radical Scavenging: 8.85 mmol TE/100 g freeze-dried mass Fremy’s Salt Scavenging: 2.15 μmol GAE/100 g freeze-dried mass	[83]
*Trifolium thalii* (flowers)	Superoxide Anion Scavenging: 1.43 μmol GAE/100 g freeze-dried mass Peroxyl Radical Scavenging: 22.45 mmol TE/100 g freeze-dried mass Fremy’s Salt Scavenging: 2.59 μmol GAE/100 g freeze-dried mass	[83]
*Trifolium longidentatum * (aerial parts)	DPPH: IC_50_ 1.71 ± 0.09 (mg/mL) for methanolic extract vs. 0.006 ± 1.10 for ascorbic acid vs. 0.016 ± 1.00 for BHT Lipid peroxidation inhibition activity: IC_50_ 41.58 ± 4.33 (μg/mL) for methanolic extract vs. 2.38 ± 0.01 for propyl gallate Trolox equivalent antioxidant capacity: 1.07 ± 0.12 mmol/l Trolox/g for methanolic extract	[84]

TE: Trolox equivalents, GAE: gallic acid equivalents, BHT: butylated hydroxytoluene.
